# Respiratory Monitoring with Textile Inductive Electrodes in Driving Applications: Effect of Electrode’s Positioning and Form Factor on Signal Quality

**DOI:** 10.3390/s25072035

**Published:** 2025-03-25

**Authors:** James Elber Duverger, Victor Bellemin, Geordi-Gabriel Renaud Dumoulin, Patricia Forcier, Justine Decaens, Ghyslain Gagnon, Alireza Saidi

**Affiliations:** 1 Institut de Recherche Robert-Sauvé en Santé et en Sécurité du Travail, Montréal, QC H3A 3C2, Canada; james.elber-duverger@irsst.qc.ca; 2 Department of Electrical Engineering, École de Technologie Supérieure, Université du Québec, Montréal, QC H3C 1K3, Canada; victor.bellemin.1@ens.etsmtl.ca (V.B.); geordi-gabriel.renaud-dumoulin.1@ens.etsmtl.ca (G.-G.R.D.); ghyslain.gagnon@etsmtl.ca (G.G.); 3 CTT Group, Saint-Hyacinthe, QC J2S 1H9, Canada; pforcier@gcttg.com (P.F.); jdecaens@gcttg.com (J.D.)

**Keywords:** respiratory monitoring, breathing sensor, textile inductive electrode, automobile, automotive application, driving application, inductive sensing, respiratory signal, signal quality index, driver health status

## Abstract

This paper provides insights into where and how to integrate textile inductive electrodes into a car to record optimal-quality respiratory signals. Electrodes of various shapes and sizes were integrated into the seat belt and the seat back of a driving simulator car seat. The electrodes covered various parts of the body: upper back, middle back, lower back, chest, and waist. Three subjects completed driving circuits with their breathing signals being recorded. In general, signal quality while driving versus sitting still was similar, compared to a previous study of ours with no body movements. In terms of positioning, electrodes on seat belt provided better signal quality compared to seat back. Signal quality was directly proportional to electrode’s height on the back, with upper back outperforming both middle and lower back. Electrodes on the waist provided either similar or superior signal quality compared to electrodes on the chest. In terms of form factor, rectangular shape outperformed circular shape on seat back. Signal quality is proportional to the size of circular electrodes on seat back, and inversely proportional to size of rectangular electrode on seat belt.

## 1. Introduction

The trend toward contactless vital signs monitoring technologies has attracted great attention in automotive innovation to improve the safety of autonomous vehicles and enable real-time assessment of driver health status [1,2]. These systems are essential for detection of a driver’s health and vigilance status, especially at some levels of autonomy where the driver must be ready to take control [3,4]. On the other hand, as traditional physiological monitoring methods, such as direct skin contact, are often unsuitable for continuous use while driving, the demand for integrated and unobtrusive systems to monitor drivers’ vital signs is constantly growing [1,5,6].

Two main physical effects—mechanical (organ boundary displacement and surface deformation) and thermal (core temperature changes through organ heat conduction)—enable unobtrusive monitoring of respiration [1,7,8]. A few studies have explored these measurement principles in automotive applications, using technologies such as radar, ballistocardiography (BCG), optical methods, inductive coupling, and thermography to detect body surface displacement or temperature [7,8]. The BCG, which records lung vibrations, offers a non-intrusive approach but suffers from motion artifacts, weak signals, noise, inconsistent sensor mechanical coupling with the driver’s body and variability in seating posture and seat designs, and seat design variability, requiring advanced signal processing and very precise sensor positioning [1,7,9]. On the other hand, radiation-based techniques, which use visible, near-infrared (NIR), and far-infrared light, face technical challenges such as line-of-sight requirements, ambient light variability on the road, and privacy concerns [1,7,8]. Infrared thermography (IRT) can detect thermal radiation to monitor temperature changes during respiration. However, this technique is strongly influenced by subject size, emotional alertness, cabin temperature variations, sunlight, reflections, and vehicle movement. As IRT only measures surface temperature, it is also sensitive to skin properties [1,8]. Radar systems detect breathing through electromagnetic phase shifts in the thorax but are subject to motion artifacts due to car vibrations, movement, and electromagnetic interference. Ultra-wideband radars must contend with bulky antennas and limited power, while higher-frequency systems reduce tissue penetration. Integrating Doppler sensors into seat belts may improve accuracy by targeting the thorax [1,6].

By way of comparison, inductive coupling systems offer significant potential for unobtrusive monitoring of respiratory activity for automotive applications, due to reduced sensitivity to ambient light and skin properties. They can be seamlessly integrated into seats or seatbelts without bulky components [10,11] and achieve good signal-to-noise ratios even during moderate vehicle movement [11,12]. This method, which uses coils generating alternating magnetic fields, detects changes in reflected impedance caused by thoracic movements, thus providing a non-invasive solution for monitoring respiration. Previous research has explored single-coil configurations powered by an oscillator (Colpitts or LC) and multi-coil configurations such as gradiometers for inductive respiration monitoring [10,11,13]. Indeed, integration of flexible and rigid single and multiple inductive coils have been explored for various applications such as mattresses [14,15], chest belts [16], or garments [16,17,18,19] for respiration monitoring.

In the automotive field, a rigid single coil integrated into the back of a car seat [11] and into the backrest of a seat placed behind an acrylic glass [10], a multi-coil gradiometer system embedded in the car seat [13], and even a flexible single coil integrated into the seat belt [20] have been evaluated for respiration detection. These types of rigid electrodes are impractical to integrate into the seat back or seat belt and can become uncomfortable for the driver. Among the various manufacturing techniques, textile inductive electrodes, though little investigated, promise seamless integration into seat back and seat belt, ensuring optimal contact with the driver. As seat covers and seat belts are generally made of fabric, embroidery is the preferred technique for integrating textile electrodes. This enables the sensors to be added directly into the finished material in a single step after the fabric has been manufactured, ensuring discreet, seamless integration [21].

Despite all these recent advances, very few studies have examined the impact of driving on the signal quality of inductive coupling detection. Only one recent study documented in the literature effectively acquired and analyzed inductive respiratory signal quality while driving a car on a road [12]. However, the focus was not on the electrodes’ positions and form factors.

In a previous paper, we introduced a quantitative method to guide the integration of textile inductive electrodes in automotive applications for respiratory monitoring [21]. A case study, with one subject sitting at rest for five minutes on a driving seat, showed the method’s ability to successfully indicate where and how to integrate the electrodes on seat belt and seat back to gather good-quality respiratory signals in an automobile.

The present study is a sequel to our previous paper, with two original features. First, respiratory signals are recorded while the subject is driving instead of sitting at rest. Second, data is analyzed for three subjects instead of one. The general question is: how can the textile inductive electrodes be integrated on seat belt and seat back to gather good-quality respiratory signals while the subject is driving? More specifically, we intend to answer the following two questions:

1. What is the effect of the electrode’s positioning on signal quality?
2. What is the effect of the electrode’s form factor on signal quality?

Answering these two questions is crucial to guide the integration of textile inductive electrodes in an automobile’s operational environment before planning on-road testing of respiratory monitoring.

## 2. Materials and Methods

### 2.1. Experimental Setup

**Figure 1:**
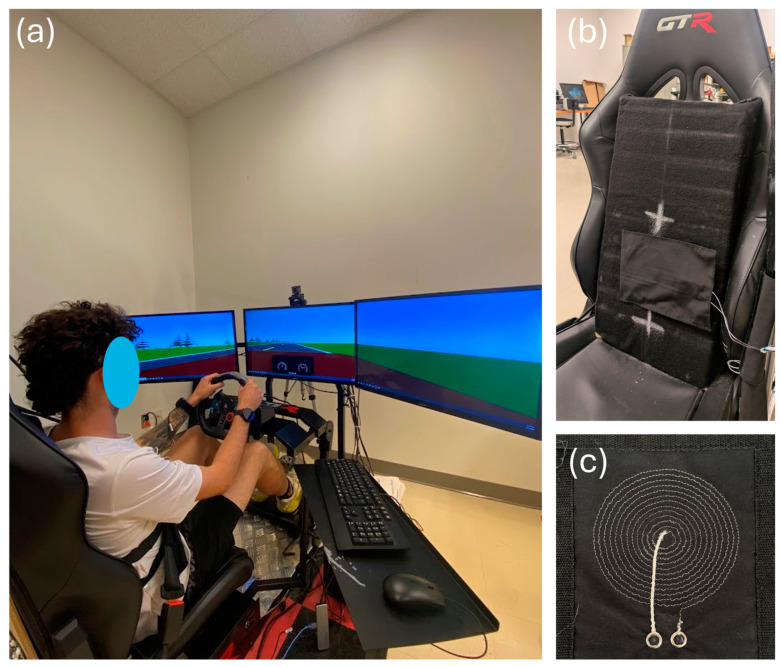
The driving simulator. (**a**) Complete setup. (**b**) Zoom-in on the car seat. The textile inductive electrode is hidden behind the black substrate fabric between the two white crosses. (**c**) The circular textile inductive electrode A.73.12.

A driving simulator (Figure 1a) was used to represent an automobile’s operational environment. The simulator was composed of the following devices:

- Three 32-inch Samsung 1080p screens—model UN32N5300AFXZC by Samsung Group (Suwon, Republic of Korea);
- A racing car seat—model S105L-BKRD by GTR Simulator (Ontario, CA, USA);
- A set of steering wheel and pedals—model B016JBE8LU by Logitech International S.A. (San Jose, CA, USA);
- A driving simulator software—York driving simulator 7.08.24 by York Computer Technologies Inc. (Kingston, ON, Canada).

Textile inductive electrodes (Figure 1b,c) were designed in the laboratory and prototyped by the CTT group (Saint-Hyacinthe, QC, Canada), a nonprofit organization which contributed to the project as a scientific partner. With either circular or rectangular design, each electrode (Table 1) was a flat coil made with a silver-plated, tin–copper alloy polyamide yarn, and embroidered onto a substrate fabric [21].

**Figure 2:**
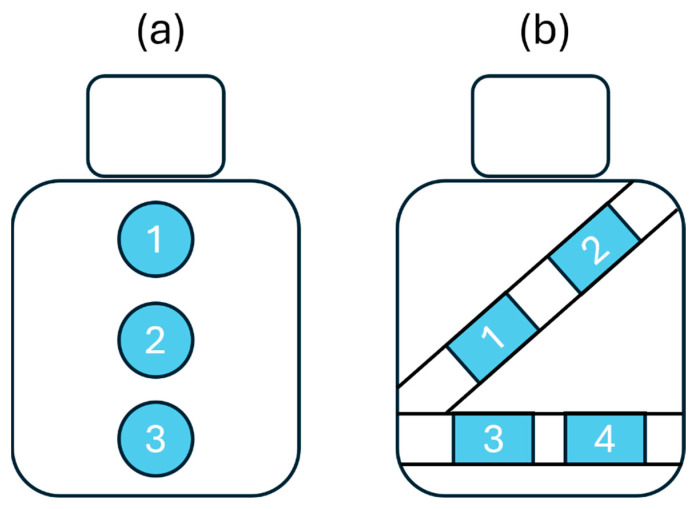
Diagram showing textile inductive electrodes’ positions. (**a**) There are three positions on the seat back: upper back (1), middle back (2), and lower back (3) position. (**b**) There are four positions on the seat belt: chest (1, 2), and waist (3, 4).

To ensure seamless integration into the vehicle, the textile inductive electrodes were directly attached to the seat back or wrapped around the seat belt with a hook and loop mechanism [21]—no action was required from the driver. As shown in the diagram of Figure 2, the electrodes were spatially distributed on the seat back and the seat belt to cover the body parts where breathing-induced displacements may be measured: upper back, middle back, lower back, chest, and waist.

### 2.2. Data Acquisition

Data was gathered on three subjects in their twenties. Only the ability to drive and body morphology were considered as inclusion criteria. Shoulder width (distance between the two acromion bones) and torso length (distance between the top of the sternum and the belly button) were measured for each subject. To characterize subject’s morphology (Table 2), the torso area was calculated as the area of the triangle with shoulder width as base and torso length as height. For the fixed experimental setup in the study, we included subjects with very different torso areas to account for a large diversity of body sizes.

Throughout every session of data acquisition, each subject was seated with seat belt on. Breathing-induced body displacements interacted with each textile electrode to generate an inductive respiratory signal that was acquired as previously described [21]. Reference respiratory signals were also independently acquired with a strain gauge around the chest [21].

**Figure 3:**
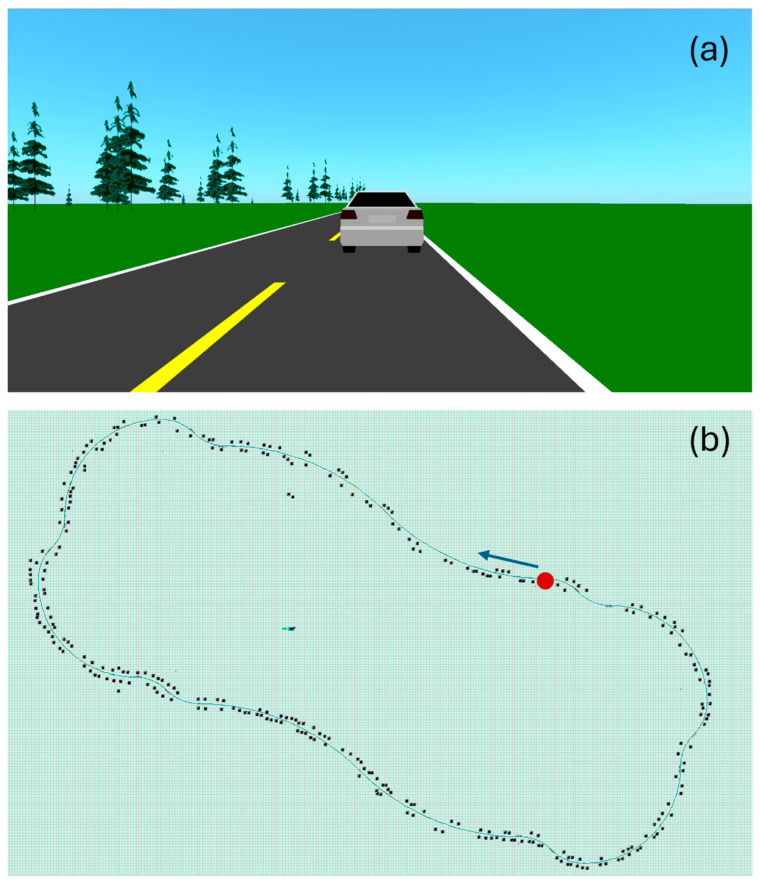
Print screens of the driving simulator’s user interface. (**a**) Scenery. The road is bordered with greenery, including trees. While driving, the subject is following a car. (**b**) Driving circuit. The green line represents the road. The black dots are trees. The circuit starts at the red dot, in the direction of the blue arrow.

For each data acquisition session, the subject was asked to follow the same driving protocol, illustrated in Figure 3. They had to follow a lead vehicle traveling at 100 km/h, alternating mostly slow curves left and right. The driving circuit was divided into 20 segments that differed by their radius of curvature (Table 3), for a total length of 14.6 km. Since driving movements are important in this study, there are no straight segments—the driver is always making micro-adjustments. To ensure homogeneity of the driving protocol from one subject to another, all subjects were asked to focus on driving—no other types of movement were allowed. It takes approximately 9.5 min to complete the circuit. A 15 min break was granted each time two circuits were completed.

Each subject completed the acquisition protocol across eight different sessions, as detailed in Table 4. For each session, the subject had to perform the complete circuit while the system recorded the following:

- One textile respiratory signal on the seat back, or else four textile respiratory signals on the seat belt (sampled at 40 Hz). On the seat belt, the four signals were acquired simultaneously.
- One reference respiratory signal from the strain gauge around the chest (sampled at 500 Hz).

According to Table 4, the number of reference signals (24) is lower compared to the number of textile signals (42) because only one reference signal is necessary for the simultaneous acquisition of 4 textile signals on seat belt (sessions 7 and 8). Indeed, the 4 textile electrodes on seat belt are located in different positions but are measuring the same sequence of the subject’s physiological breathing and therefore need only one independent reference signal in the same timeframe.

### 2.3. Data Analysis

The calculations in this study were performed with MatlabR2024b^®^. All respiratory signals were pre-processed as previously described [21]. Each respiratory signal was then segmented into 34 sections of one minute using a moving window of 60 s sliding with a step of 15 s. On each one-minute section of signal, four noise-based signal quality indexes (SQIs) were calculated, as previously explained [21]:

- SBR (signal-to-baseline ratio). Higher SBR value means lower effect of baseline wandering on signal quality.
- SHR (signal-to-high-frequency-noise ratio). Higher SHR value means lower effect of high frequency noise on signal quality.
- MMR (median-to-mean ratio). Higher MMR value means lower effect of motion artifact on signal quality.
- SPC (spectral correlation) between textile and reference spectra. Higher SPC value means the textile-based signal is as likely as the reference signal to allow breathing rate calculation through peak detection.

To make a general assessment of signal quality throughout the study, the textile electrode signals were compared to reference signals. For each signal, the 34 SBR values were averaged, leading to one SBR value per signal. The same process was repeated for SHR and MMR. The textile and reference SQIs were pooled in two groups, and the null hypothesis was tested with the two-sided Wilcoxon rank sum test, with the significant *p* value at 0.05.

To compare the signal quality of a textile group A versus a textile group B, we first make a common pool of the SBR values of the two groups and normalize between 0 and 1. The same process is repeated for SHR, MMR, and SPC. Then, a signal quality metric (SQM) that combines the SQIs is calculated via the geometric mean:

(1) $$SQM=\sqrt{SBR\;.\;SHR\;.\;MMR\;.\;SPC}$$

The SQM is then normalized between 0 and 1, with the best signal receiving the score of 1, and the poorest signal the score of 0. The common pool of SQM values is then dispatched between the two groups A and B. The null hypothesis is tested with the two-sided Wilcoxon rank sum test, with the significant *p* value at 0.05.

## 3. Results

### 3.1. General Assessment of Signal Quality

**Figure 4:**
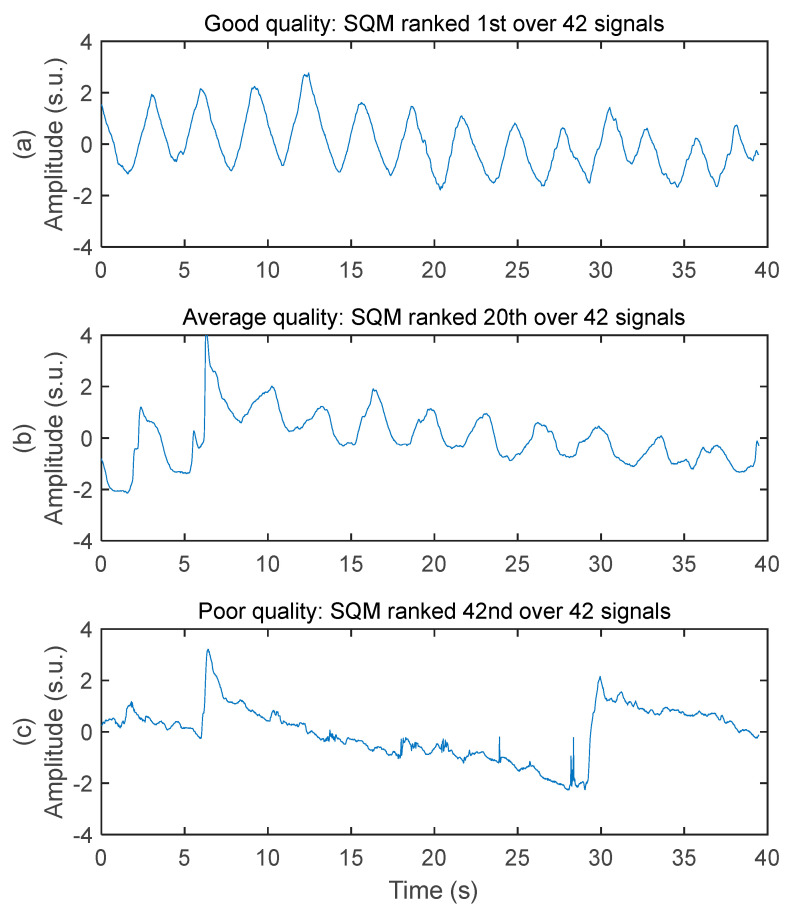
Examples of respiratory signal with various signal quality. Amplitudes are all in standard score unit and displayed within the same axis limits to allow direct comparison. (**a**) Good signal quality. Recorded on the chest of subject #1 with a 50 cm^2^ rectangular electrode on seat belt. (**b**) Average signal quality. Recorded on the chest of subject #3 with a 50 cm^2^ rectangular electrode on seat belt. (**c**) Poor signal quality. Recorded on the middle back of subject #3 with a 28 cm^2^ circular electrode on seat back.

The system acquired respiratory signals of various quality while the subjects were driving. Three samples of decreasing signal quality are illustrated in Figure 4. The good-quality signal has virtually no baseline wandering, no high frequency noise, no motion artifact, and very good ability to allow peak detection for breathing rate calculation. The average-quality signal has moderate baseline wandering, very low level of high frequency noise, moderate level of motion artifact (see the abrupt change of amplitude between 5 and 10 s), and good ability to allow peak detection for breathing rate calculation. The poor-quality signal has high-amplitude baseline wandering, low level of high frequency noise, high-amplitude motion artifact, and poor ability to allow peak detection for breathing rate calculation.

**Figure 5:**
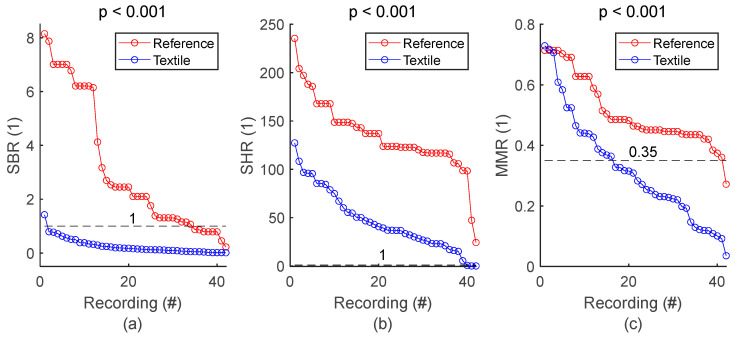
General assessment of signal quality: reference vs. textile. (**a**) Comparison of SBR values. (**b**) Comparison of SHR values. (**c**) Comparison of MMR values. SQIs are sorted in decreasing order and plotted for reference and textile electrodes. There is one dot for each respiratory signal. The value of one dot is the average of 34 SQIs calculated on 34 one-minute sections of signal.

SQI values from all respiratory signals and corresponding statistics are shown in Figure 5 and Table 5, respectively. SBR of textile signals was mostly below 1, indicating respiratory signals with lower power compared to baseline, and therefore baseline wandering challenging signal quality. SHR values mostly higher than 1 indicated respiratory signals with higher power compared to high frequency noise, meaning that type of noise had low impact on signal quality. Furthermore, visual inspection of the whole set of textile respiratory signals showed motion artifact started to challenge signal quality at MMR values of 0.35. In fact, more than half of the 42 textile-based respiratory signals had noticeable motion artifacts. However, episodes of motion artifacts were transient and did not prevent the rest of the signal from being of good quality. Figure 5 and Table 5 also demonstrated lower signal quality for textile electrodes compared to reference strain gauge, with lower SBR, SHR and MMR values, all with significant *p* values. There was only one reference signal for 4 textile signals simultaneously acquired on seat belt, leading to 42 textile signals against 24 reference signals. For the sake of clarity, reference SQI values for seat belt in Figure 5 are repeated in the graph to have a total of 42 values. However, the *p* value in both Figure 5 and Table 5 is calculated with the original 24 reference values.

### 3.2. Signal Quality on the Seat Back

#### 3.2.1. Signal Quality vs. Electrode’s Position on Seat Back

**Figure 6:**
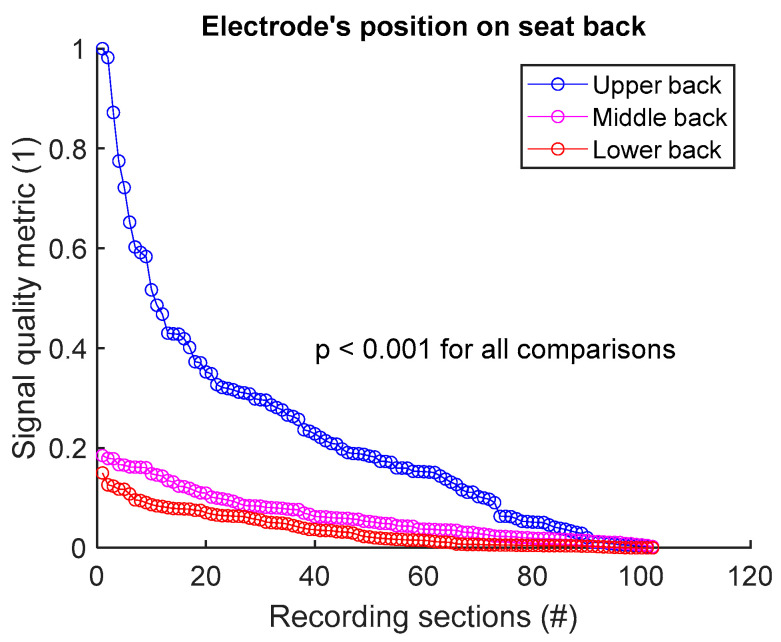
Signal quality vs. electrode’s position on seat back. Normalized SQMs are sorted in decreasing order and plotted. There is one dot for each one-minute section of textile respiratory signal. The value of one dot is the SQM calculated for one section of respiratory signal.

To assess signal quality as a function of electrode’s position on seat back, signals from sessions #1, #2, and #3 (Table 4) were analysed for the three subjects. Normalized SQM values were dispatched into three groups: upper back, middle back, and lower back. Results are shown in Figure 6 and Table 6. Signal quality was directly proportional to electrode’s height on the back. In fact, Upper back outperformed both middle and lower back. Middle back displayed better outcome compared to lower back. All observed differences were statistically significant.

#### 3.2.2. Signal Quality vs. Electrode’s Size on Seat Back

**Figure 7:**
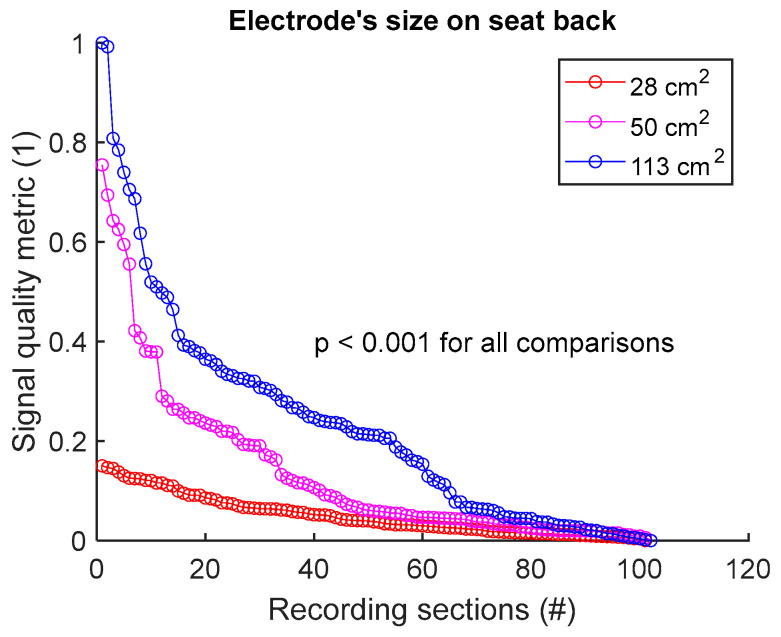
Signal quality vs. electrode’s size on seat back. Normalized SQMs are sorted in decreasing order and plotted. There is one dot for each one-minute section of textile respiratory signal. The value of one dot is the SQM calculated for one section of respiratory signal.

To assess signal quality as a function of electrode’s size on seat back, signals from sessions #2, #4, and #5 (Table 4) were analysed for the three subjects. Normalized SQM values were dispatched into three groups: 28 cm^2^, 50 cm^2^, and 113 cm^2^, corresponding to the surface areas of circular electrodes. Results are shown in Figure 7 and Table 7. Signal quality was directly proportional to electrode’s size. In fact, 113 cm^2^ outperformed both 50 cm^2^ and 28 cm^2^. Furthermore, 50 cm^2^ displayed better outcome compared to 28 cm^2^. All differences were statistically significant.

#### 3.2.3. Signal Quality vs. Electrode’s Shape on Seat Back

**Figure 8:**
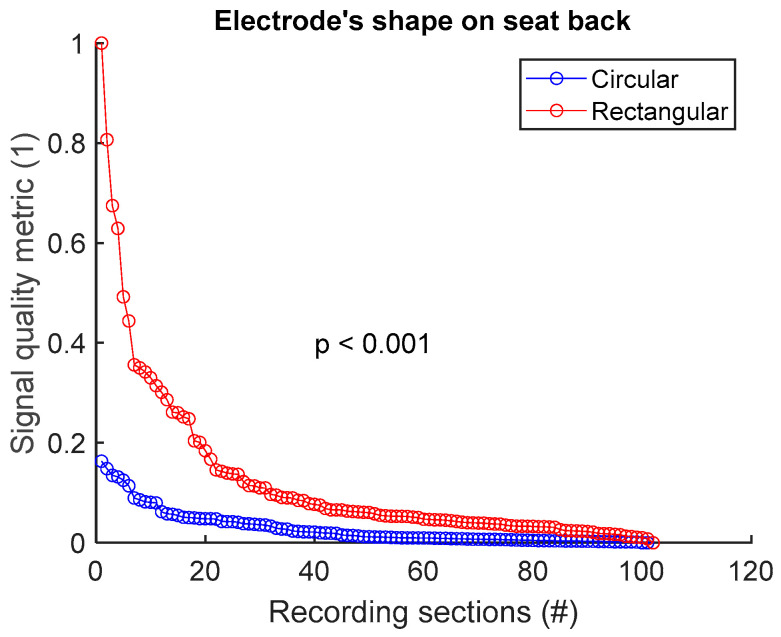
Signal quality vs. electrode’s shape on seat back. Normalized SQMs are sorted in decreasing order and plotted. There is one dot for each one-minute section of textile respiratory signal. The value of one dot is the SQM calculated for one section of respiratory signal.

To assess signal quality as a function of electrode’s shape on the seat back, signals from sessions #4 and #6 (Table 4) were analysed for the three subjects. Normalized SQM values were dispatched into two groups: circular shape and rectangular shape. Results are shown in Figure 8 and Table 8. Rectangular shape outperformed circular shape. The difference between the two groups was statistically significant.

### 3.3. Signal Quality on the Seat Belt

#### 3.3.1. Signal Quality vs. Electrode’s Position on Seat Belt

**Figure 9:**
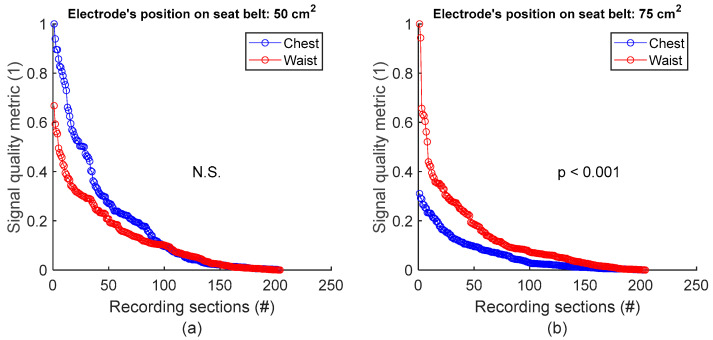
Signal quality vs. electrode’s position on seat belt. (**a**) Chest vs. waist with 50 cm^2^ electrode. (**b**) Chest vs. waist with 75 cm^2^ electrode. For each panel, normalized SQMs are sorted in decreasing order and plotted. There is one dot for each one-minute section of textile respiratory signal. The value of one dot is the SQM calculated for one section of respiratory signal.

To assess signal quality as a function of electrode’s position on seat belt, signals from session #8 (Table 4), with 50 cm^2^ surface area electrodes, were first analysed for the three subjects. Normalized SQM values were dispatched into two groups: chest and waist. Results are shown in Figure 9a and the left side of Table 9. The difference between the chest and waist groups was not statistically significant. However, the same analysis using session #7 (Table 4), with 75 cm^2^ surface area electrodes, demonstrated that waist outperformed chest with statistically significant difference (see Figure 9b and the right side of Table 9).

#### 3.3.2. Signal Quality vs. Electrode’s Size on Seat Belt

**Figure 10:**
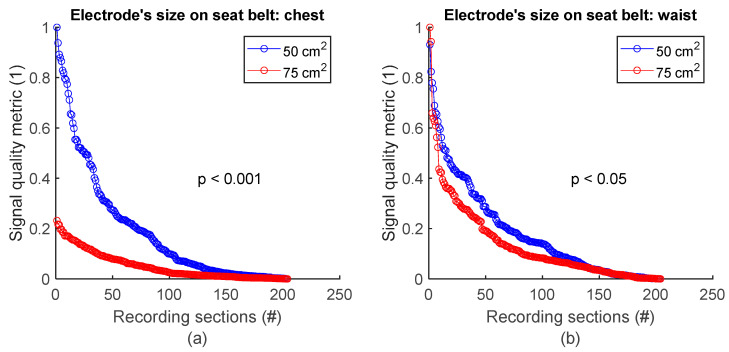
Signal quality vs. electrode’s size on seat belt. (**a**) 50 cm^2^ vs. 75 cm^2^ on chest. (**b**) 50 cm^2^ vs. 75 cm^2^ on waist. Normalized SQMs are sorted in decreasing order and plotted. There is one dot for each one-minute section of textile respiratory signal. The value of one dot is the SQM calculated for one section of respiratory signal.

To assess signal quality as a function of electrode’s size on the seat belt, chest signals (Figure 2b) from sessions #7 and #8 (Table 4) were analysed for the three subjects. Normalized SQM values were dispatched into two groups according to electodes’ surface area: 50 cm^2^ and 75 cm^2^. Results are shown in Figure 10a and the left side of Table 10. On the chest, 50 cm^2^ electrodes outperformed 75 cm^2^ ones. The difference between the two groups was statistically significant. Similarily, the same analysis using waist signals (Figure 2b) from sessions #7 and #8 (Table 4) demonstrated 50 cm^2^ electrodes also outperformed 75 cm^2^ ones with statistically significant difference (see Figure 10b and the right side of Table 10).

### 3.4. Signal Quality: Electrode on Seat Back vs. Electrode on Seat Belt

**Figure 11:**
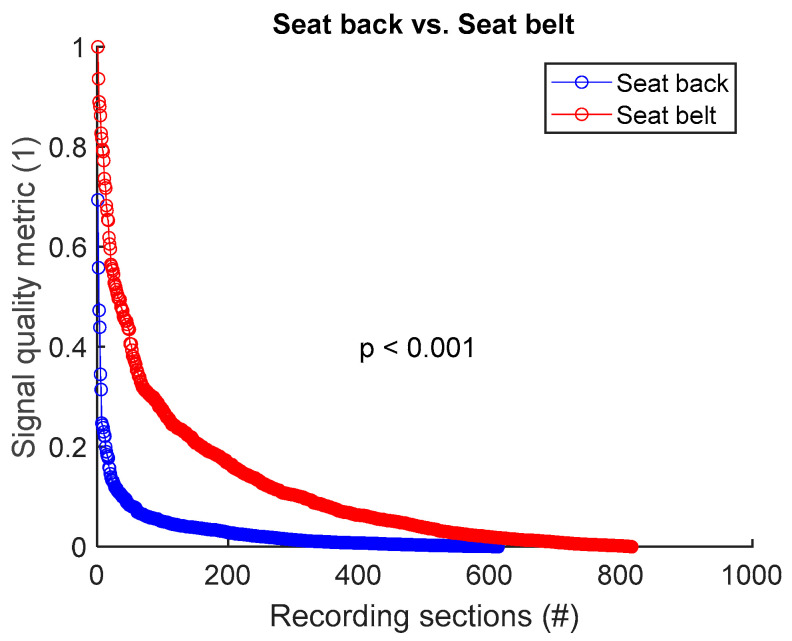
Signal quality: seat back vs. seat belt. Normalized SQMs are sorted in decreasing order and plotted. There is one dot for each one-minute section of textile respiratory signal. The value of one dot is the SQM calculated for one section of respiratory signal.

To assess signal quality as a function of electrode’s position on seat back versus seat belt, signals from all 8 sessions (Table 4) were analysed for the three subjects. Normalized SQM values were dispatched into two groups: seat back and seat belt. Results are shown in Figure 11 and Table 11. Electrode placement on seat belt outperformed the seat back positioning. The difference between the two groups was statistically significant.

## 4. Discussion and Conclusions

The goal of this paper was to gain insights into the optimal integration of textile inductive electrodes in a car, for achieving good-quality respiratory monitoring while the subject is driving. To isolate the specific context of driving-induced body movements, a simulator was used instead of an on-road approach, and the subjects were asked to focus on driving and nothing else. Signals were then acquired, and the effects of the electrode’s positioning and form factor on signal quality was assessed. To our knowledge, this study is the first to perform such an assessment.

The overall signal quality while driving was similar to the quality observed while sitting still [21]. Indeed, high-frequency noise was still low, and baseline wandering was not more challenging compared to a static setting. Interestingly though, motion artifacts started to be challenging at MMR = 0.35, compared to MMR = 0.2 in our previous study [21], which suggests more motion artifacts while driving. However, driving movements have no impact on our previously stated design rules on where and how to integrate textile electrodes to acquire good-quality signals [21]. Whether the subject was sitting at rest [21] or driving in the present study, seat belt still provided better signal quality compared to seat back. Upper back still granted better signal quality compared to lower back. Interestingly, waist electrodes provided better signals compared to chest electrodes depending on the size.

We therefore went further than our previous study [21] on quantifying the impact of form factors on signal quality. As observed, geometry and size matter. A rectangular shape outperformed a circular shape on seat back. Signal quality was proportional to the size of circular electrodes on seat back, and inversely proportional to the size of rectangular electrode on seat belt. This emphasizes the importance of form factors, even if they are less impactful compared to electrode positions, as demonstrated by our previous study [21]. Further investigations could help to bring more insights into the effect of electrodes’ form factors on signal quality. To ensure generalizable results, those investigations should notably cover complex shapes and extreme sizes, and also study the combined effects of shapes and sizes.

The study involved three subjects, but we expect the results to be applicable to a wider population and not to be influenced by the specific characteristics of each subject. The textile inductive electrode detects breathing-induced body displacements to provide a signal and only requires to be close to or to be deformed by a human body [21]. Characteristics like health, age, and sex do not matter. The only criterion that counts for a specific subject is where the electrodes are located on the body. As a matter of fact, larger breathing-induced body displacements yield better respiratory signal quality—that is why, for instance, signal quality is better on the chest compared to the lower back, independently of the subject. Therefore, if the setup is fixed, the subject’s body size may matter if the electrodes do not fall in the same places. However, we selected subjects with very different body sizes and observed no impact on the general trends in the results. If the seat back and the seat belt fit the body size, the electrodes will cover the same body parts, and we expect the results to be similar. Furthermore, for a specific body position, signal quality varies with electrode’s size and shape because form factors may influence body–electrode interactions and not because of the specific characteristics of a subject.

In our previous study, as well as the present one, we focused on the respiratory rate, as an essential parameter for detecting events such as drowsiness in vehicles. The impact of motion artifacts on the signals is less of a concern, as the main objective is not medical, but event detection. Nevertheless, continuous recording is essential, even in the event of temporary loss of signals due to motion artifacts. Some designs place rigid inductive electrodes inside the car seat, but increased distance from the body reduces signal amplitude, affecting accuracy. Textile electrodes were chosen in our study for their seamless integration into the seat belt and their higher signal amplitude, essential for respiratory rate detection. Hence, our approach could minimize the compromise between signal quality and measurement accuracy.

The driving movements and driving circuits in this study are representative of what would happen during normal driving on a highway, with some limitations. First, there is no road-induced vibration and no g-force on a driving simulator, and therefore no noise related to these phenomena. Second, drivers may have more non-driving-related body movements in real life. Finally, the driving wheel diameter of the driving simulator is smaller compared to standard driving wheels and the wheel was also more sensitive according to subjects’ feedback. Consequently, arm movements with lower amplitudes were needed for driving, and hence they may have had less impact on signal quality. However, it is important to note that at 100 km/h (62 miles/h), the amplitude of arms movements is small anyway. Another study, with the subjects driving on a road, with vehicle vibration and exposure to temperature and humidity variations, could help address these limitations.

In conclusion, this paper gave us some insights into where and how to integrate textile inductive electrodes into a car to acquire good-quality respiratory signals while the subject is driving. Similarly to when the subject is sitting still, the best signal quality came from the seat belt compared to the seat back, with subject’s waist and upper back providing better signal than subject’s chest and lower back, respectively. Electrode’s size and shape also modulate signal quality in a non-trivial way that deserves further investigations. We have demonstrated that textiles inductive electrodes, integrated with our design guidelines [21], could be suitable candidates for testing reliable acquisition of good-quality respiratory signals on smooth roads like highways.

## Figures and Tables

**Table 1:** Types of electrodes. For circular designs, the dimension is the diameter. Inductances were measured by an Instek LCR916 handheld LCR meter at 100 kHz (Good Will Instrument Co., Ltd., Taipei, Taiwan).

Prototype	Shape	Dimension (mm)	Area (cm^2^)	Number of Turns	Inductance (µH)
A.73.12	Circular	60	28	10	2.9
A.73.15	Circular	120	113	20	19.8
A.73.20	Circular	80	50	13	5.8
A.73.39	Rectangular	150 × 50	75	5	2.5
A.73.40	Rectangular	100 × 50	50	5	1.7

**Table 2:** Morphology of the subjects.

Subject (#)	Sex	Shoulder Width (cm)	Torso Length (cm)	Torso Area (cm^2^)
1	Male	55	52	1430
2	Male	44	40	840
3	Female	36	38	684

**Table 3:** Driving circuit segmentation.

Segment (#)	1	2	3	4	5	6	7	8	9	10
Radius (m)	2000	2000	400	400	850	400	400	850	400	400
Arc (m)	1531	1531	306	306	1202	306	306	1202	306	306
Direction	Right	Left	Right	Left	Left	Right	Left	Left	Right	Left
**Segment (#)**	**11**	**12**	**13**	**14**	**15**	**16**	**17**	**18**	**19**	**20**
Radius (m)	2000	2000	400	400	850	400	400	850	400	400
Arc (m)	1531	1531	306	306	1202	306	306	1202	306	306
Direction	Right	Left	Right	Left	Left	Right	Left	Left	Right	Left

**Table 4:** Acquisition protocol followed by each subject. The subject performed the complete circuit 8 times, once for each acquisition session.

Session (#)	Host	Electrode	Position	Area (cm^2^)	Shape	Number of Textile Signals	Number of Reference Signals
1	Seat back	A.73.12	Upper back (1)	28	Circular	1	1
2	Seat back	A.73.12	Middle back (2)	28	Circular	1	1
3	Seat back	A.73.12	Lower back (3)	28	Circular	1	1
4	Seat back	A.73.20	Middle back (2)	50	Circular	1	1
5	Seat back	A.73.15	Middle back (2)	113	Circular	1	1
6	Seat back	A.73.40	Middle back (2)	50	Rectangular	1	1
7	Seat belt	A.73.39	Chest and Waist (1, 2, 3, 4)	75	Rectangular	4	1
8	Seat belt	A.73.40	Chest and Waist (1, 2, 3, 4)	50	Rectangular	4	1
**Note.** For the three subjects, the complete set of recordings provided 42 textile respiratory signals and 24 reference respiratory signals.							

**Table 5:** General assessment of signal quality. The number of samples n is the number of signals, meaning that a unique averaged SQI value was calculated per signal.

	SBR (1)		SHR (1)		MMR (1)	
	Textile	Reference	Textile	Reference	Textile	Reference
Median	0.26	2.98	47	134	0.32	0.50
Mean	0.16	2.60	43	129	0.32	0.50
STD	0.28	1.52	16	26	0.09	0.08
Range	1.08	5.89	58	105	0.32	0.31
n	42	24	42	24	42	24
*p*	3.57 × 10^−10^		3.98 × 10^−9^		5.85 × 10^−5^	

**Table 6:** Signal quality vs. electrode’s position on seat back. The number of samples n is the number of one-minute sections of signal: 102 sections = 3 subjects × 1 signal per subject × 34 sections per signal.

	Upper Back SQM (1)	Middle Back SQM (1)	Lower Back SQM (1)
Median	0.178	0.050	0.020
Mean	0.228	0.063	0.036
STD	0.214	0.049	0.036
Range	0.998	0.183	0.150
n	102	102	102
*p* value	Upper vs. middle: *p* = 2.38 × 10^−11^ Upper vs. lower: *p* = 4.03 × 10^−18^ Middle vs. lower: *p* = 2.13 × 10^−6^		

**Table 7:** Signal quality vs. electrode’s size on seat back. The number of samples n is the number of one-minute sections of signal: 102 sections = 3 subjects × 1 signal per subject × 34 sections per signal.

	28 cm^2^ SQM (1)	50 cm^2^ SQM (1)	113 cm^2^ SQM (1)
Median	0.037	0.059	0.212
Mean	0.049	0.137	0.233
STD	0.040	0.164	0.221
Range	0.150	0.755	1.000
n	102	102	102
*p* value	28 cm^2^ vs. 50 cm^2^: *p* = 2.46 × 10^−5^ 28 cm^2^ vs. 113 cm^2^: *p* = 1.24 × 10^−12^ 50 cm^2^ vs. 113 cm^2^: *p* = 5.18 × 10^−4^		

**Table 8:** Signal quality vs. electrode’s shape on seat back. The number of samples n is the number of one-minute sections of signal: 102 sections = 3 subjects × 1 signal per subject × 34 sections per signal.

	Circular Shape SQM (1)	Rectangular Shape SQM (1)
Median	0.011	0.056
Mean	0.028	0.123
STD	0.035	0.169
Range	0.163	1.000
n	102	102
*p* value	3.19 × 10^−15^	

**Table 9:** Signal quality vs. electrode’s position on seat belt. The number of samples n is the number of one-minute sections of signal: 204 sections = 3 subjects × 2 signals per subject × 34 sections per signal.

	Electrodes on Belt: 50 cm^2^		Electrodes on Belt: 75 cm^2^	
	Chest SQM (1)	Waist SQM (1)	Chest SQM (1)	Waist SQM (1)
Median	0.093	0.097	0.026	0.070
Mean	0.190	0.131	0.062	0.131
STD	0.232	0.137	0.071	0.161
Range	1.000	0.668	0.311	1.000
n	204	204	204	204
*p* value	0.213		8.81 × 10^−7^	
**Important note**: SQM values for 50 cm^2^ and 75 cm^2^ shall not be compared since they have not been normalized in a common pool.				

**Table 10:** Signal quality vs. electrode’s size on seat belt. The number of samples n is the number of one-minute sections of signal: 204 sections = 3 subjects × 2 signals per subject × 34 sections per signal.

	Electrodes on Belt: Chest		Electrodes on Belt: Waist	
	50 cm^2^ SQM (1)	75 cm^2^ SQM (1)	50 cm^2^ SQM (1)	75 cm^2^ SQM (1)
Median	0.096	0.022	0.136	0.080
Mean	0.191	0.050	0.183	0.136
STD	0.229	0.055	0.189	0.160
Range	1.000	0.232	0.931	1.000
n	204	204	204	204
*p* value	7.91 × 10^−13^		1.76 × 10^−2^	
**Important note**: SQM values for chest and waist shall not be compared since they have not been normalized in a common pool.				

**Table 11:** Signal quality: seat back vs. seat belt. The number of samples n is the number of one-minute sections of signal. 612 samples on seat back = 3 subjects × 6 signals per subject × 34 sections per signal. 816 samples on seat belt = 3 subjects × 8 signals per subject × 34 sections per signal.

	Electrodes on Seat Back SQM (1)	Electrodes on Seat Belt SQM (1)
Median	0.013	0.062
Mean	0.032	0.120
STD	0.059	0.156
Range	0.694	1.000
n	612	816
*p* value	3.09 × 10^−58^	

## Data Availability

The data for the project were collected at the Electrical Engineering Department, École de Technologie Supérieure. For ethical and confidentiality reasons, the authors cannot provide public access to them. Nevertheless, the authors agree to make data and materials supporting the results or analyses available for the investigation of scientific integrity if necessary.
